# The economic burden of chronic disease care faced by households in Ukraine: a cross-sectional matching study of angina patients

**DOI:** 10.1186/1475-9276-12-38

**Published:** 2013-05-30

**Authors:** Adrianna Murphy, Ajay Mahal, Erica Richardson, Andrew E Moran

**Affiliations:** 1 European Centre on Health of Societies in Transition, Department of Health Services Research and Policy, London School of Hygiene and Tropical Medicine, 15-17 Tavistock Place, London WC1H 9SH, UK; 2 Department of Medicine, Nursing and Health Sciences, Monash University, Victoria 3800, Australia; 3 European Observatory on Health Systems and Policies, London School of Hygiene and Tropical Medicine, 15-17 Tavistock Place, London WC1H 9SH, UK; 4 Division of General Medicine, Columbia University Medical Center, 622 W. 168th Street, PH 9 East, Room 105, New York, NY 10032, USA

**Keywords:** Chronic disease/economics, Health expenditure, Health services accessibility, Angina pectoris, Ukraine

## Abstract

**Introduction:**

Non-communicable diseases (NCDs) are the leading cause of death and disability worldwide, and their prevalence in lower- and middle-income countries (LMIC) is on the rise. The burden of chronic health expenditure born by patient households in these countries may be very high, particularly where out-of-pocket payments for health care are common. One such country where out-of-pocket payments are especially high is Ukraine. The financial impact of NCDs on households in this country has not been researched.

**Methods:**

We set out to explore the burden of NCD care in Ukraine with a study of angina patients. Using data from the Ukraine World Health Survey of 2003 we employed the novel Coarsened Exact Matching approach to estimate the difference in out-of-pocket payment (OPP) for health care between households with a stable angina pectoris (a chronic form of IHD) patient and those without. The likelihood of engaging in catastrophic spending and using various distress financing mechanisms (e.g. sale of assets, borrowing) among angina households compared with non-angina households was also explored.

**Results:**

Among angina patient households (n = 203), OPP occupied an average of 32% of household effective income. After matching, angina households experienced significantly higher monthly per capita OPP for health care (B = $2.84) and medicines (B = $2.94), but were not at significantly higher odds of engaging in catastrophic spending. Odds of engaging in ‘sale of assets’ (OR = 2.71) and ‘borrowing’ (OR = 1.68) to finance OPP were significantly higher among angina households.

**Conclusions:**

The cost of chronic care in Ukraine places a burden on individual patient households. Households of angina patients are more likely to engage in distress financing to cover the cost of treatment, and a high proportion of patients do not acquire prescribed medicines because they cannot afford them. This warrants further research on the burden of NCD care in other LMIC, especially where OPP for health care is common. Health policies aimed at reducing OPP for health care, and especially medicines, would lessen the high health and financial burden of chronic care. Further research is also needed on the long-term impact of borrowing or sale of assets to finance OPP on patient households.

## Introduction

The epidemic of non-communicable diseases (NCDs) that is occurring in lower- and middle- income countries (LMIC) is well documented [1,2]. In 2008, nearly 80% of deaths from NCDs occurred in LMIC, caused mostly by ischemic heart disease (IHD) [3].

What is less understood is how these countries will manage the burden of the health costs associated with treating NCDs, which require different care models to those of communicable diseases. For example, among patients diagnosed with IHD, acute events and hospitalization or death can be avoided, but only with a daily drug regimen and long-term medical attention. As a result, management of IHD and other chronic NCDs may impose a heavy financial burden on the households of uninsured patients. A recent study showed that more than 50% of IHD patient households in China, India and Tanzania engaged in catastrophic health spending (≥ 40% of non-food expenditures) due to out-of-pocket payments (OPP) for health care, and many were turning to distress financing (borrowing, sale of assets) to cover these costs [4].

The impact of health care costs for NCDs may be especially important in countries of the former Soviet Union (fSU), which experience the highest rate of disability-adjusted life years due to IHD globally, and where health care systems are struggling to reform under great economic and human resource constraints. High OPP for health care in these countries have been reported [5-9] but there have been no studies of the financial burden attributable to IHD specifically. We set out to explore the impact of IHD in one fSU country, Ukraine, using a case study of angina patient households. Ukraine has been relatively ignored in the public health literature, despite evidence of a high prevalence of NCD risk factors in the country [10,11] and a health care system that has proved unable to meet the needs of its patients [9,12].

Officially, all Ukrainians are entitled to a guaranteed package of health care services provided free of charge at the point of use; however, resource constraints have led to attempts by the government to limit the range of services covered in this package. With few exceptions, outpatient pharmaceuticals have never been covered in this package. Only 0.5% of Ukraine’s population is covered by voluntary insurance schemes [13]. In the absence of pre-payment for care, OPP for drugs and medicines, service charges, and informal payments to medical personnel have increased, placing the financial burden on the shoulders of the patient and their household [12]. The share of private households’ OPP in total health expenditure in Ukraine is estimated at about 40.5%, higher than in neighbouring Belarus (17.4%) and Russia (28.3%) [14]. A recent analysis of cross-sectional survey data from Ukraine indicated that 95.5% of respondents who accessed care in the last four weeks reported making an OPP; the median OPP when accessing outpatient care was $12.57 USD, $62.84 USD for inpatient care and $18.85 USD for pharmaceuticals [9].

In Ukraine where, in 2009, 16.1% of the population did not earn a living wage [12], and 14.4% of the population live below the national poverty line, it is likely that the effect of OPP for IHD on patient households is great [12,15]. An earlier analysis of the 1996 Income Expenditure Survey found that approximately 3.9% of households in Ukraine engage in catastrophic health spending for health care generally [8]; however, this study did not address alternative mechanisms used by households to finance OPP, such as borrowing and sale of assets, which are common methods of coping with OPP in other countries [16-22]. Failure to consider such coping mechanisms can lead to an overestimate of the effect of OPP on household consumption in the short-term (because it ignores the increase in income from alternative sources), but an underestimate of the longer term impoverishing impact from indebtedness or the loss of returns on assets and savings [20,22].

We set out to estimate the impact of OPP for IHD care on households of patients with angina (one form of IHD) in Ukraine using data from the World Health Survey 2003 (WHS). In order to account for other household characteristics that may influence household health care spending we employed the novel ‘Coarsened Exact Matching’ approach [28].

## Methods

### Data

WHS data with no individual participant identifiers are publicly available ( http://www.who.int/healthinfo/survey/en/index.html). The WHS sampling procedure is described in detail elsewhere [23]. In brief, all Ukrainian men and women ≥18 years of age in every geographic region of Ukraine, who were not out of the country during the survey period (July/August 2003), were eligible for sampling. The WHS consisted of an individual questionnaire and a household questionnaire. Our analysis included only those who answered both, resulting in a final sample size of n = 2860. Only one respondent was selected from each household using Kish tables (a method used for random selection). Women were over-sampled (approx. 65%) compared with men (approx. 35%). Sampling weights were included in the WHS data and were used in this analysis. All WHS participants provided informed consent prior to study participation.

### Angina definition

Because the WHS did not ask specifically about IHD history (only non-specific “heart disease” history), we chose to analyze respondents who reported stable angina pectoris, or typical exertional chest pain, a common sequela of IHD. Past international studies have shown that angina prevalence is strongly associated with non-fatal myocardial infarction prevalence and other forms of IHD [24]. Our angina patient sample included those respondents who reported the following: history of physician diagnosis and treatment of angina, and a diagnosis of ‘definite angina’ based on the Rose questionnaire [24]. Physician diagnosis and treatment of angina were defined by affirmative responses to both of the questions: “Have you ever been diagnosed with angina or angina pectoris (a heart disease)?” and “Have you ever been treated for it?” Definite angina based on the Rose questionnaire was defined by typical affirmative answers to all Rose questionnaire questions. The Rose questionnaire, sometimes referred to as the London School of Hygiene Cardiovascular Questionnaire, was developed as a “standard, unbiased and validated measure of the prevalence of angina in general populations” [24]. Details on the adaptation of the Rose questionnaire to the Ukraine WHS data can be found in Additional file 1: Table S1.

### Household expenditure and financing sources

Expenditures were reported by respondents in Ukrainian hryvnia and converted into United States dollars (USD) using the 2003 exchange rate. Monthly per capita total household expenditure was determined by each respondent’s self-report of total household spending in the last four weeks, divided by the reported number of people living in the household. The specific question used in the WHS was: “In the last four weeks, how much did your household spend in total?” Monthly per capita health care expenditure was determined from each respondent’s self-report of total health care costs in the last four weeks, less any insurance reimbursements, divided by the household size. The specific question used in this analysis was: “In the last four weeks, how much did your household spend on health care costs, less any insurance reimbursements?”. Monthly household expenditure on medicines was determined using the respondent’s self-reported household spending on medicines in the last four weeks (“In the last four weeks how much did your household spend on medication or drugs?”). For all three of these measures, extreme outliers were managed using ‘top-coding’ i.e. reducing their values to that of the 99th percentile (a method that has been used previously [25]. We opted not to use the aggregate of self-reported itemized health spending (which was also included in the WHS) because in the WHS generally, the average of this measure is higher than the average reported total health spending [26] and, therefore, using the reported total produces in a more conservative estimate of health spending. We acknowledge that this may result in a higher estimate of the share of health spending occupied by spending on medicines than had we used the aggregate of itemized health spending. Effective monthly income was determined by subtracting monthly subsistence spending (defined as the average reported food spending in the last four weeks of those households in the 45th–55th percentile of food spending, accounting for household size (approximately $21.87 USD per person in the household,)) from each household’s self-reported total monthly household expenditure [8]. This approach was used rather than subtracting each household’s reported food spending from their reported total spending because there is evidence that in poorer households, food expenditure occupies a larger share of total expenditure [27]; therefore, it is preferable to estimate subsistence expenditure based on the average food spending of households in the 45–55 percentile of food spending. This method was used in a previous multi-country study of catastrophic health spending [8]. Households were classified as engaging in catastrophic spending if monthly health care costs exceeded 40% of effective income. Financing sources used by households in the last year to cover health care costs were determined by the respondents’ response to the question: “In the last 12 months, which of the following financial sources did your household use to pay for any health expenditures?”.

### Coarsened exact matching

A crude comparison of mean household expenditure and the likelihood of engaging in catastrophic spending between households with and without an angina patient would ignore the fact that there may be other characteristics of angina households that are driving health care expenditure, such as the number of people or the proportion of elderly living in the household. In order to account for this, we employed the novel ‘coarsened exact matching’ method (CEM) [28]. The CEM method has been described in detail elsewhere [28,29]. Briefly, CEM attempts to control for the potential confounding influence of ‘pre-treatment’ covariates on the outcome of interest, by matching ‘treatment’ cases with ‘non-treatment’ cases that are approximately similar to them with regard to those covariates. CEM has an advantage over other methods of matching observational data such as propensity-score matching (PSM) and exact matching (EM) in that it doesn’t require that the matched observations are balanced in terms of pre-treatment covariates as does PSM, nor does it require matched observations to be precisely similar in terms of these covariates as in EM [28,30]. Instead, CEM ‘coarsens’ the pre-treatment covariates into categories, based on their distribution or on natural or intuitive divisions [28,30]. In our case, ‘treatment’ cases are angina households and ‘non-treatment’ controls are non-angina households. We used CEM to account for the potential confounding influence of the following pre-treatment household characteristics on household expenditure: i) household size ii) level of education attained by the head of the household (to account for the socio-economic status of the household) iii) proportion of females in the household (to account for the generally higher health expenditure associated with older men) iv) proportion of people over the age of 60 in the household v) proportion of women over the age of 60 in the household (to account for the longer life expectancy and therefore possibly higher health care costs of women in Ukraine) vi) proportion of children under the age of five in the household and vii) self-report of arthritis, asthma, depression, schizophrenia/psychosis or diabetes (to account for co-morbidities that might affect chronic health care costs). After matching, we used linear regression to analyse the difference in mean per capita household net health spending, health care expenditure and expenditure on medicines associated with angina households and logistic regression to analyse the odds of catastrophic spending and of using various financing mechanisms among angina households, compared with non-angina households.

## Results

### Angina prevalence

More than 90% of the Ukraine WHS sample responded to the angina-related survey questions. Using our definition of angina, we found an estimated prevalence of 5.5% among men and 8.0% among women (age ≥18 years old; N = 203). Prevalence of angina by gender and age group are shown in Table 1. Angina prevalence was highest in the 70+ age group for both men and women (15.8% for both).

**Table 1:** Number and proportion of angina patients by age and gender, WHS-Ukraine 2003

***Age(years)***	Males (n = 1003*)	Females (n = 1847*)	***Total (n = 2860)***
*18-39*	2 (0.5%)	6 (0.9%)	8 (0.8%)
*40-69*	35 (7.2%)	99 (10.8%)	134 (9.6%)
*70+*	18 (15.8%)	43 (15.8%)	61 (15.4%)
***Total***	**55 (5.5%)**	**148 (8.0%)**	**203 (7.1%)**

* Male and Female columns do not add up to total due to missing data on gender for 10 observations.

### Household expenditure and financing mechanisms

Table 2 summarizes household expenditure among angina households (for those that responded to the given questions), as well as the proportion of angina households engaged in catastrophic health spending. Over 91% of the sample responded to the question about total household health expenditure. Of those reporting requiring health care in the last 30 days 85% responded to the question about total household health expenditure in the last four weeks and of those reporting being prescribed medicines, 79% responded to the question about expenditure on medicines specifically.

**Table 2:** Expenditure and catastrophic spending among angina and non-angina households in Ukraine, WHS 2003, in 2003 United States Dollars (USD), crude, unmatched analysis

	**Angina**	**Non-angina**
*Per capita monthly household expenditure (USD)*	*Mean (S.E.)*	
*All health*	9.26 (2.75)	6.25 (0.43)
*Medicines only*	7.99 (2.37)	4.92 (0.66)
*Effective income*	29.09 (4.93)	26.76 (1.09)
	*Proportion (%)*	
*Catastrophic health spending*	44.2	35.1

Expenditure on health generally occupied approximately 32% of effective income, while spending on medicines specifically occupied approximately 27%. Almost half of angina households were classified as engaging in catastrophic health spending (44.2%). Table 2 presents these figures for non-angina households as well (before matching).

**Figure 1:**
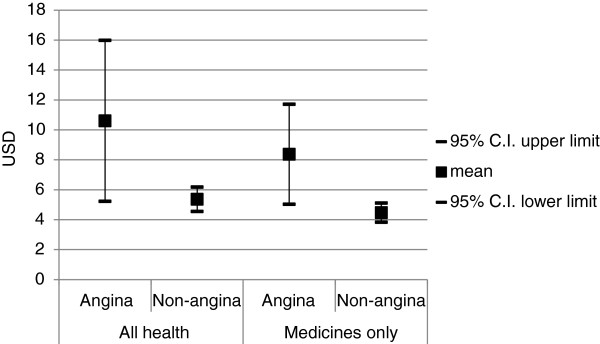
Mean monthly per capita OPP for all health care and medicines only, angina (n=139) and non-angina (n=1106) matched households, WHS-Ukraine 2003.

Using the CEM approach we were able to match 139 angina households to non-angina households; the following analyses include only those that were matched and that responded to the relevant questions. Absolute differences in mean monthly per capita spending on health, and on medicines specifically, between angina households and non-angina households are presented in Figure 1. After matching, angina households spent an average of $5.24 (p = 0.001) more on health and $3.90 (p < =0.001) more on medicines compared with non-angina households (Figure 1). Per capita monthly non-medical spending did not differ significantly between the two groups (B = −$3.63, p = 0.295).

Odds ratios (OR) in Table 3 represent the odds of catastrophic spending, as well as the odds of financing OPP from each listed source, among angina households compared with non-angina households. After matching, angina households had 1.36 times the odds of catastrophic health spending of non-angina households, though this result was not statistically significant. The odds of financing OPP using all listed mechanisms were all higher among angina patients, but only statistically significantly so for ‘sale of assets’ (OR = 6.23) (Table 3). Angina patients were almost four times more likely to miss work due to ill health than non-angina respondents.

**Table 3:** Likelihood of engaging in catastrophic spending, means of financing OPP and missing work among angina households compared to non-angina households after matching, WHS-Ukraine 2003

	***OR***	***S.E.***	***P-value***
*Catastrophic spending*	1.21	0.22	0.285
*Means of financing OPP:*			
*Income*	1.62	0.44	0.075
*Savings*	1.02	0.40	0.959
*Sale of assets*	6.23	3.49	0.001
*Borrowing from friends or family*	1.42	0.33	0.130
*Borrowing from others*	1.11	0.41	0.777
Missing work due to ill health*	3.71	2.34	0.038

*This outcome was assessed using the individual level survey, comparing angina respondents to non-angina respondents.

## Discussion

Using a conservative case definition, we found an angina prevalence of 7.1% in Ukrainian adults aged ≥18 years from a population-based sample, which is at the higher end of the angina prevalence range observed in surveys from around the world [31]. High angina prevalence fits with IHD mortality rates in Ukraine and the rest of Eastern Europe, which are among the highest globally [32]. Our estimate of angina prevalence from the WHS Ukraine is similar to previous estimates for Ukraine (7.3%) [33], Russia (6.5%) [31] and Georgia (9.4%) [34] for the same age range, and the female-to-male ratio (approx. 45% higher) is consistent with the reported average 20% higher prevalence of angina in women [31].

The proportion of total household effective income dedicated to OPP for health care in angina patient households in the Ukraine WHS is much higher than the national average reported by the State Statistics Committee in 2002 (approximately 3.2%) [35], and the largest share of this is spent on medicines. This finding should be interpreted with caution, as it has been noted that the WHS generally produces higher estimates of health expenditure than other surveys [26]. This may occur because 1) the intensive health focus of the WHS may cause respondents to include health spending that occurred prior to the recall period, 2) the recall period used in the WHS (four weeks) differs from that used in other expenditure surveys, or 3) the WHS was conducted later than other surveys (2002–2003) and health spending may have actually increased in the WHS countries over that period of time [26]. The WHS 2003 Ukraine Report itself estimates that approximately 20% of the sample population experienced catastrophic health spending [36]. This is lower than our estimate of 35% in the non-angina sample; however, although in both cases catastrophic health spending was defined as health expenditure exceeding 40% of effective income, where we defined subsistence spending (used to calculate effective income) as the average expenditure on food of those households in the 45th–55th percentile of food expenditure (approximate $59 USD), as done in previous research on the subject [8,27,37], the WHS Ukraine Report defines subsistence spending as the poverty line (approximately $50 USD). A lower estimate of subsistence spending results in a higher estimate of effective income which in turn reduces the estimated proportion of households whose health expenditure will reach the catastrophic threshold. This highlights the sensitivity of estimates of catastrophic spending to differences in definition of subsistence and effective income.

Our estimates of catastrophic health expenditure are also higher than those from previous research in Georgia (11.7%) [37], another fSU country, which used the same method of calculating subsistence spending. This may be partly due to the fact that a higher proportion of Georgians do not seek care when they are ill (61%) compared to Ukrainians (52%) [9], or to the fact that the question in the Ukraine WHS that we used to measure health expenditure (i.e. ‘total self-reported household health expenditure) may have captured health expenditures which might not be included in the Georgian study (such as health care products and alternative healers). Moreover, the data used in the Georgian study is from 2009 (the WHS 2003 Georgia Report estimated catastrophic spending at about 26% [38]), and it is possible that newer data from Ukraine would also reflect a reduction in OPP for health care. However, research shows that prices for drugs in Ukraine have increased due to the 2008 economic crisis [39] and, according to data from 2010, 70% of Ukrainians diagnosed with hypertension were not taking recommended drug treatment [40].

Despite the difference between our estimate and previous estimates from the region, the levels of OPP for health care observed in angina households, and in particular the OPP for medicines, are cause for concern and should serve as impetus for further research on the financial burden faced by angina patients in other formerly Soviet countries where IHD rates are high and OPP are common [9]. Our estimates relate only to chronic, stable angina and not acute hospitalizations for myocardial infarction or ischemic heart failure. As these other IHD conditions are likely experienced by many of the angina patients in our sample, our estimates may only represent the ‘tip of the iceberg’ in terms of the economic consequences of IHD in Ukraine. A recent study conducted in four other low- and middle-income countries (LMIC) reported high rates of catastrophic spending among households of patients hospitalized for an acute IHD event [4]. Although regulated by the Ukrainian government, prices for pharmaceuticals have increased in the country due to inflation and lack of control over prescribing practices – 14% of the 28% increase in pharmaceutical prices observed in the country between 2006 and 2008 was related to doctors prescribing more expensive medicines instead of cheaper ones at various stages of treatment [12].

Our analysis of matched data indicated that households of angina patients may experience higher OPP costs for health care relative to non-angina households. The higher OPP observed for medicines specifically is not surprising, given that treating angina often requires a long-term (sometimes life-long) daily medication regimen. The OPP costs of medications that treat angina, as well as other chronic cardiovascular conditions such as hypertension or heart failure, may provide one possible explanation for the irregular treatment observed in Ukraine (73.5% not taking prescribed daily medication) [40]. In fact, in the Ukraine WHS data, of those angina patients who reported that the reason for their latest visit to a doctor was ‘heart disease’, approximately 62% did not obtain all medicines prescribed to them because they ‘could not afford’ them (author’s calculations). This is particularly worrisome given the importance of adherence to a standard daily drug regimen as a means of reducing the likelihood of acute coronary event [41].

Although almost half of angina households were defined as engaging in catastrophic spending, after adjustment for matching their odds of being so were not significantly higher, nor was their non-medical consumption significantly lower than non-angina households. This suggests the catastrophic spending measure may over-estimate the short-term impact of high OPP on household consumption. One reason for this observation may be that angina households are partly financing their consumption by borrowing or selling assets, thus ‘protecting’ their consumption from the potentially catastrophic effects of OPP. Indeed, the likelihood of selling assets to finance OPP was significantly higher among angina households in the Ukraine WHS. Engaging in these types of coping mechanisms has been shown to disguise the longer-term impoverishing effects of OPP [20], but the extent of this impoverishment may depend on the character and interest rates imposed by the lender [22,42], as well as whether any assets sold were essential to current consumption or surplus to requirements [17]. Some previous qualitative work suggests that although such coping mechanisms solve immediate cash flow problems, over the long-term households may be forced to spend less on education [43], reduce food or treatment quality or return to work before fully recovered [44] in order to meet financial obligations. Further qualitative or longitudinal research is needed in order to understand the consequences of these coping mechanisms in the Ukrainian context.

The impoverishing effects of angina on households in Ukraine might also be underestimated here because we are not able to measure the *indirect* effects of ill health on household finances. In particular, we found that angina patients were almost four times as likely to miss work due to ill health as matched respondents without angina; lost wages among these patients, especially if they are the primary wage-earner in the home, may have significant long-term effects on household finances that cannot be measured in this survey. In the case of farming households, the lost productivity of the ill household member may be difficult to substitute, especially in poorer or smaller households [18].

### Limitations

Use of the Rose Questionnaire to diagnose angina may lead to a high rate of false positive angina diagnoses among women [45]. This is of particular concern in the present study, in which approximately 73% of angina patients were female. However, by restricting our angina definition to those who also self-reported a physician diagnosis and treatment of angina—a definition that correlates well with prevalence of acute myocardial infarction survivors and IHD mortality [24] —we made every effort to account for this potential limitation.

This analysis relies on self-reported data on household spending, which is subject to measurement error [46,47]. Respondents may provide answers that they believe are socially acceptable and when asked to provide frequencies or amounts, they may rely on best estimates rather than recalling and counting [48]. The results of a test-retest study of the WHS specifically found that respondents in this survey tended to under-report total household expenditure, and over-report out-of-pocket health expenditure [26]. This pattern, in addition to the reasons for high estimates of health spending in the WHS discussed above, may explain the discrepancy between the WHS estimates of monthly household out-of-pocket spending on health and those reported by the State Statistics Committee of Ukraine. Nonetheless, regarding our comparative analyses, there is no reason to expect that within the WHS, self-report should be less accurate in angina compared with non-angina patients.

Lastly, this analysis relies on WHS data from 2003 and more recent data would provide a more accurate estimate of the burden of OPP faced by angina patients in Ukraine. However, WHO indicators from 2008 show that private OPP continue to occupy a large percentage of total health expenditure in the country [14], and prices for drugs in Ukraine have increased due to the 2008 economic crisis [39], suggesting that significant improvements in OPP for chronic health care since 2003 are unlikely.

## Conclusion

As the population of Ukraine and other fSU countries continues to age, and smoking rates among young adults remains high, the prevalence of NCDs is likely to increase in the future. The cost of managing chronic disease currently places an burden on households of patients. Our results suggest that OPP for health care, especially for medicines, are higher in angina households and that these expenditures are often financed by borrowing or the sale of assets. The potentially detrimental effect of such coping mechanisms on households’ livelihoods and the national economy in Ukraine, as well as in other fSU countries where OPP are high, requires further research. Along with increased funding of preventive public health programs, the burden of health care costs due to NCDs should be addressed by financing mechanisms which improve access to outpatient prescription pharmaceuticals as well as stricter regulation and oversight of prescribing and dispensing practices.

## Competing interests

The authors declared that they have no competing interest.

## Authors’ contributions

ADM was responsible for the conception and design of the study, the analysis and interpretation of its results, drafting the manuscript and revising it critically. She has given final approval of the version to be published. AJM contributed to the conception and design of the study, the interpretation of its results and the critical revision of the manuscript. He has given final approval of the version to be published. ER contributed to the interpretation of the study results and the critical revision of the manuscript. She has given final approval of the version to be published. ANM contributed to the conception and design of the study, the interpretation of its results, and the critical revision of the manuscript. He has given final approval of the version to be published. All authors read and approved the final manuscript.

## Supplementary Material

Additional file 1: Table S1 WHS modified Rose angina questionnaire. Survey participants were asked about symptoms experienced within the prior 12 months. Click here for file

## References

[B1] Leeder S Raymond S Greenberg H Liu H Esson K A Race against time: The challenge of cardiovascular disease in developling countries 2004 New York: Trustees of Columbia University

[B2] WHO Global burden of disease: 2004 update 2004 Geneva: WHO

[B3] WHO Global status report on non-communicable diseases 2010 2010 Geneva: WHO

[B4] Huffman MD Rao KD Pichon-Riviere A Zhao D Harikrishnan S Ramaiya K Ajay VS Goenka S Calcagno JI Caporale JE A cross-sectional study of the microeconomic impact of cardiovascular disease hospitalization in four low- and middle-income countries PLoS One 2011 6 e20821 10.1371/journal.pone.0020821 21695127 PMC3114849

[B5] Belli P Gotsadze G Shahriari H Out-of-pocket and informal payments in health sector: evidence from Georgia Health Policy 2004 70 109 123 10.1016/j.healthpol.2004.03.007 15312713

[B6] Falkingham J Poverty, out-of-pocket payments and access to health care: evidence from Tajikistan Soc Sci Med 2004 58 247 258 10.1016/S0277-9536(03)00008-X 14604611

[B7] Skarbinski J Walker HK Baker LC Kobaladze A Kirtava Z Raffin TA The burden of out-of-pocket payments for health care in Tbilisi, Republic of Georgia JAMA 2002 287 1043 1049 10.1001/jama.287.8.1043 11866656

[B8] Xu K Evans DB Kawabata K Zeramdini R Klavus J Murray CJ Household catastrophic health expenditure: a multicountry analysis Lancet 2003 362 111 117 10.1016/S0140-6736(03)13861-5 12867110

[B9] Balabanova D Roberts B Richardson E Haerpfer C McKee M Health care reform in the former Soviet Union: beyond the transition Health Serv Res 2012 47 840 864 10.1111/j.1475-6773.2011.01323.x 22092004 PMC3419892

[B10] Webb CP Bromet EJ Gluzman S Tintle NL Schwartz JE Kostyuchenko S Havenaar JM Epidemiology of heavy alcohol use in Ukraine: findings from the world mental health survey Alcohol Alcohol 2005 40 327 335 10.1093/alcalc/agh152 15824065

[B11] Gilmore AB McKee M Telishevska M Rose R Epidemiology of smoking in Ukraine, 2000 Prev Med 2001 33 453 461 10.1006/pmed.2001.0915 11676587

[B12] Lekhan V Rudiy V Richardson E Ukraine: health system review Health Syst Transit 2010 12 1 183 21429862

[B13] World Development Indicatorshttp://data.worldbank.org/indicator

[B14] National Health Accounts: Ukrainehttp://www.who.int/nha/country/ukr/en/

[B15] Cherenko L Living standards in Ukraine 2006 Kyiv: Konsultant Publishing

[B16] McPake B Hanson K Mills A Community financing of health care in Africa: an evaluation of the Bamako initiative Soc Sci Med 1993 36 1383 1395 10.1016/0277-9536(93)90381-D 8511627

[B17] Russell S Ability to pay for health care: concepts and evidence Health Policy Plan 1996 11 219 237 10.1093/heapol/11.3.219 10160370

[B18] Sauerborn R Adams A Hien M Household strategies to cope with the economic costs of illness Soc Sci Med 1996 43 291 301 10.1016/0277-9536(95)00375-4 8844932

[B19] McIntyre D Thiede M Dahlgren G Whitehead M What are the economic consequences for households of illness and of paying for health care in low- and middle-income country contexts? Soc Sci Med 2006 62 858 865 10.1016/j.socscimed.2005.07.001 16099574

[B20] Flores G Krishnakumar J O’Donnell O van Doorslaer E Coping with health-care costs: implications for the measurement of catastrophic expenditures and poverty Health Econ 2008 17 1393 1412 10.1002/hec.1338 18246595

[B21] Leive A Xu K Coping with out-of-pocket health payments: empirical evidence from 15 African countries Bull World Health Organ 2008 86 849 856 10.2471/BLT.07.049403 19030690 PMC2649544

[B22] Wagstaff A Measuring Financial Protection in Health 2008 Washington, DC: The World Bank

[B23] The World Health Survey (WHS) Sampling guidelines for participating countries 2002 http://www.who.int/healthinfo/survey/whssamplingguidelines.pdf

[B24] Mathers C Truelsen T Begg S Satoh T Global burden of ischaemic heart disease in the year 2000 2004 Geneva: World Health Organization

[B25] Levine D Polimeni R Ramage I Insuring Health or Insuring Wealth? An experimental evaluation of health insurance in rural Cambodia Impact Analyses Series 2012 Paris: Agence Francais de Developpement

[B26] Xu K Ravndal F Evans DB Carrin G Assessing the reliability of household expenditure data: results of the World Health Survey Health Policy 2009 91 297 305 10.1016/j.healthpol.2009.01.002 19217184

[B27] Deaton A Muellbauer J Economics and consumer behaviour 1980 Cambridge: Cambridge Universiy Press

[B28] Iacus S King G Porro G Causal inference without balance checking: coarsened exact matching Polit Anal 2012 20 1 24 10.1093/pan/mpr013

[B29] Blackwell M Iacus S King G Porro G Cem: coarsened exact matching in Stata Stata J 2010 9 524 546

[B30] Fan VY Mahal A What prevents child diarrhoea? The impacts of water supply, toilets, and hand-washing in rural India J Dev Effect 2011 3 340 370 10.1080/19439342.2011.596941

[B31] Hemingway H Langenberg C Damant J Frost C Pyorala K Barrett-Connor E Prevalence of angina in women versus men: a systematic review and meta-analysis of international variations across 31 countries Circulation 2008 117 1526 1536 10.1161/CIRCULATIONAHA.107.720953 18347213

[B32] Mortality country fact sheet Ukraine 2006

[B33] World Bank What underlies Ukraine’s Mortality Crisis? Health and Demography 2010 World Bank

[B34] Balabadze M Chumburidze I Bakradze N Tataradze R Possibility of using WHO questionnaires, distributed by mail, for detecting stenocardia druing mass screening [in Russian] Kardiologiia 1989 29 42 44 2531249

[B35] Ukraine State Statistics Committee Household income and expenditure 2004 Kiev 2004 2009

[B36] WHO World Health Survey 2003: Report of Ukraine 2004 Geneva: WHO

[B37] Gotsadze G Zoidze A Rukhadze N Household catastrophic health expenditure: evidence from Georgia and its policy implications BMC Health Serv Res 2009 9 69 10.1186/1472-6963-9-69 19400939 PMC2695816

[B38] WHO WHS 2003 Survey: Report of Georgia 2004 Geneva: WHO

[B39] Mladovsky P Srivastava D Cylus J Karanikolos M Evetovits T Thomson S McKee M Health policy responses to the financial crisis in Europe 2012 Geneva: WHO; European Observatory on Health Systems and Policies 28837306

[B40] Roberts B Stickley A Balabanova D Haerpfer C McKee M The persistence of irregular treatment of hypertension in the former Soviet Union J Epidemiol Community Health 2012 60 1079 1082 22447959 10.1136/jech-2011-200645

[B41] Boden WE O’Rourke RA Teo KK Hartigan PM Maron DJ Kostuk WJ Knudtson M Dada M Casperson P Harris CL Optimal medical therapy with or without PCI for stable coronary disease N Engl J Med 2007 356 1503 1516 10.1056/NEJMoa070829 17387127

[B42] Coping with the costs of severe illness in rural China http://www.ids.ac.uk/files/Wp58.pdf

[B43] Waddington C Enyimayew K A price to pay: the impact of user charges in ashanti-akim district, Ghana Int J Health Plann Manage 1989 4 17 47 10.1002/hpm.4740040104

[B44] Kabir MA Rahman A Salway S Pryer J Sickness among the urban poor: a barrier to livelihood security J Int Dev 2000 12 707 722 10.1002/1099-1328(200007)12:5<707::AID-JID703>3.0.CO;2-G

[B45] Garber CE Carleton RA Heller GV Comparison of “rose questionnaire angina” to exercise thallium scintigraphy: different findings in males and females J Clin Epidemiol 1992 45 715 720 10.1016/0895-4356(92)90048-R 1619450

[B46] Visaria P Poverty and living standards in Asia Popul Dev Rev 1980 6 189 223 10.2307/1972728

[B47] Anand S Harris C Choosing a welfare indicator Am Econ Rev 1994 84 226 231

[B48] Kimberlin CL Winterstein AG Validity and reliability of measurement instruments used in research Am J Health Syst Pharm 2008 65 2276 2284 10.2146/ajhp070364 19020196

