# Supplementary material for: The economic burden of chronic disease care faced by households in Ukraine: a cross-sectional matching study of angina patients
Source: Int J Equity Health. 2013 May 30;12:38. doi: 10.1186/1475-9276-12-38 (PMC3691525; doi:10.1186/1475-9276-12-38)
Supplement: Additional file 1: Table S1 — WHS modified Rose angina questionnaire. Survey participants were asked about symptoms experienced within the prior 12 months. [file 1475-9276-12-38-S1.doc]

Additional file 1: Table S1: WHS modified Rose angina questionnaire. Survey participants were asked about symptoms experienced within the prior 12 months.

| 1 | Pain or discomfort in your chest when you walk uphill or hurry? |
| --- | --- |
| 2 | Pain or discomfort in your chest when you walk at an ordinary pace on level ground? |
| 3 | What do you do if you get it while you are walking? ? *(Affirmative response = “stop or slow down” or “carry on after taking a pain relieving medicine that dissolves in your mouth”)* |
| 4 | If you stand still, what happens to it? *(Affirmative response = “relieved”)* |
| 5 | Where is the pain? *(Affirmative response = upper or middle chest, lower chest, or left arm)* |
| Definite angina was defined as affirmative answers to either question one or two and specified answers to all of questions three to five. Past studies, performed predominantly with male subjects, showed the Rose questionnaire had 78-81% sensitivity and 94-97% specificity in identifying angina, using physician diagnosis as the standard. (19) Although other validation studies suggest the Rose questionnaire can lead to a high rate of false positive diagnoses in women compared with men (21), Rose questionnaire-diagnosed angina in women has been associated with risk factors for and sub-clinical signs of IHD,(22-24) and IHD mortality.(25-29) In order to verify that the angina sample was representative of IHD patients in general, we also conducted analyses in WHS participants reporting use of inpatient or outpatient medical services for “heart disease” within the prior 12 months (WHS Questions 7003 &7403). Of these heart disease patients, 35% were also in the angina sample. | |
